# Is it really possible to leave sars-cov-2 outside the door? Bianca Maria Bocci

**DOI:** 10.1093/eurpub/ckac131.072

**Published:** 2022-10-25

**Authors:** BM Bocci, I De Palma, D Amodeo, G Cevenini, N Nante, G Messina

**Affiliations:** 1 Post Graduate School of Public Health, University of Siena, Siena, Italy; 2 Department of Medical Biotechnologies, University of Siena, Siena, Italy; 3 Department of Molecular and Developmental Medicine, University of Siena, Siena, Italy

## Abstract

**Background:**

In this historical period, it has become very important to live in healthy environments. By using everyday objects, cross-contamination is possible because of prolonged microbial persistence on surfaces. UV-C irradiation is an environmentally friendly method to disinfect objects as no harmful chemicals or heat are involved. This study aims to determine the virucidal activity, against SARS-CoV-2, of UV-C irradiation occurring in a designed UV device, ‘Purity Capsule'.

**Methods:**

An experimental study was performed in September 2020. The ‘Purity capsule’ has an 11 W lamp (3.5W UV-C) positioned in the centre of the device. The lamp has a dome covered with a reflective, protective coating. Three metal carriers were placed at the maximum distance from the UV-C lamp in three different positions and tested at 30 and 60 seconds 3 times. The carriers were inoculated with 100 µL of SARS-CoV-2 viral suspension with a concentration of 106.5 TCID50 /mL. After treatment, laboratory procedures were used to transfer the treated virus from carriers to multiwell plates. The samples were compared with positive controls (not exposed to UV-C light) after incubation, at 37 °C in 5% COÕ · in a humidified atmosphere, for 3 days. The residual viral activity was tested by assessing the 50% infectious dose per tissue culture (TCID50%).

**Results:**

Tests performed at 30 seconds of UV-C irradiation show an average viral reduction of 4.0 Log10 (99.99%). All three tests performed at 60 seconds reached the maximum measurable log10 viral reduction: 5.0 Log10 (99.999%).

**Conclusions:**

The study assessed the effectiveness of the device in significantly reducing the viral load on all carriers regardless exposure time and distance from the UV-C light source, with no impact on the level of environmental pollution.

**Key messages:**

• UV-C light has the property of inactivating viral growth; its physical approach is considered a good compromise between cost and effectiveness.

• The device was effective in disinfecting all small everyday objects tested.

